# Dinuclear gold(I) Complexes with Bidentate NHC Ligands as Precursors for Alkynyl Complexes via Mechanochemistry

**DOI:** 10.3390/molecules27134317

**Published:** 2022-07-05

**Authors:** Valentina Stoppa, Edoardo Battistel, Marco Baron, Paolo Sgarbossa, Andrea Biffis, Gregorio Bottaro, Lidia Armelao, Cristina Tubaro

**Affiliations:** 1 Dipartimento di Scienze Chimiche, Università degli Studi di Padova, via F. Marzolo 1, 35131 Padova, Italy; valentina.stoppa@studenti.unipd.it (V.S.); edoardo.battistel@studenti.unipd.it (E.B.); andrea.biffis@unipd.it (A.B.); lidia.armelao@unipd.it (L.A.); 2 CIRCC-Consorzio per le Reattività Chimiche e la Catalisi, c/o Dipartimento di Scienze Chimiche, Università degli Studi di Padova, via F. Marzolo 1, 35131 Padova, Italy; paolo.sgarbossa@unipd.it (P.S.); gregorio.bottaro@cnr.it (G.B.); 3 Dipartimento di Ingegneria Industriale, Università degli Studi di Padova, via F. Marzolo 9, 35131 Padova, Italy; 4 ICMATE-CNR, c/o Dipartimento di Scienze Chimiche, Università degli Studi di Padova, via F. Marzolo 1, 35131 Padova, Italy; 5 Dipartimento di Scienze Chimiche e Tecnologie dei Materiali, Consiglio Nazionale delle Ricerche, Piazzale A. Moro 7, 00185 Roma, Italy

**Keywords:** gold complexes, NHC ligands, alkynyl complexes, bidentate ligands, photophysical properties

## Abstract

The use of alkynyl gold(I) complexes covers different research fields, such as bioinorganic chemistry, catalysis, and material science, considering the luminescent properties of the complexes. Regarding this last application, we report here the synthesis of three novel dinuclear gold(I) complexes of the general formula [(diNHC)(Au-C≡CPh)_2_]: two Au-C≡CPh units are connected by a bridging di(N-heterocyclic carbene) ligand, which should favor the establishment of semi-supported aurophilic interactions. The complexes can be easily synthesized through mechanochemistry upon reacting the pristine dibromido complexes [(diNHC)(AuBr)_2_] with phenylacetylene and KOH. Interestingly, we were also able to isolate the monosubstituted complex [(diNHC)(Au-C≡CPh)(AuBr)]. The gold(I) species were fully characterized by multinuclear NMR spectroscopy and mass spectrometry. The emission properties were also evaluated, and the salient data are comparable to those of analogous compounds reported in the literature.

## 1. Introduction

Alkynyl gold(I) complexes represent an interesting class of compounds [1] which can find application in different research fields, such as bioinorganic chemistry [2,3,4] or catalysis [5,6,7,8]. Among the possible applications, special attention has been dedicated to the development of highly emissive species [9]. The luminescence properties of these compounds in solution are generally associated with the organic part of the molecule and are associated with LLCT or LMCT transitions, although, in the solid state, the emission properties also depend on the self-assembly of the molecular units. Furthermore, π–π stacking involving the organic part, as well as Au···Au interactions, play a fundamental role in the isolation of different supramolecular structures or polymorphs, characterized by different emitting properties [10,11,12,13,14].

The linear dicoordination in the L-Au-C≡CR fragment is granted by the coordination of neutral ligands, L, such as phosphines, or more recently, N-heterocyclic carbene ligands [15,16]. In this frame, reports on alkynyl gold(I) complexes with bridging dicarbene ligands are still limited in the literature [12,17], despite the possible presence of an intramolecular aurophilic interaction that can influence the emitting properties [18].

**Scheme 1:**
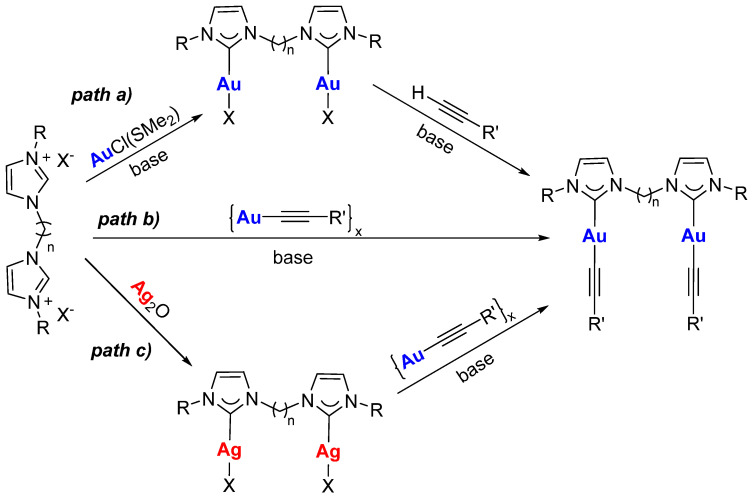
Different synthetic pathways for the synthesis of [(diNHC)(Au-C≡CR)_2_] complexes.

The complexes with bridging dicarbene ligands can be isolated via three different strategies (Scheme 1): (a) synthesis of the dinuclear complex with the bridging dicarbene ligand and halides in the coordination sphere, followed by halide substitution with alkynyl moieties in the presence of a base (for example K_2_CO_3_ or Tl(acac)) [12,17]; (b) reaction of the bis(imidazolium) salt with the gold(I) acetylide polymeric species in the presence of a base (for example, Cs_2_CO_3_) [12]; (c) transmetalation of the dicarbene ligand from the silver(I) dinuclear complex to the gold(I) acetylide polymeric species [17].

In this report, we have explored the mechanochemical synthesis of the dinuclear [(diNHC)(Au-C≡CR)_2_] complexes, starting from the corresponding dibromido complexes and phenylacetylene. The complexes differ in the length of the linker between the carbene units and the nitrogen wingtip substituents.

## 2. Results

**Scheme 2:**
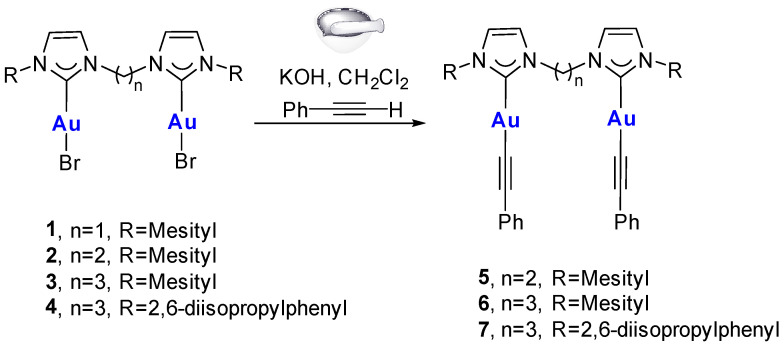
Synthesis of complexes **5**–**7**.

The synthesis of the new alkynyl gold(I) complexes **5**–**7** was performed via a ligand substitution reaction, i.e., by reacting the pristine complexes having bromide ligands with an excess of phenylacetylene (Au:PhC≡CH ratio 1:10) and potassium hydroxide (Scheme 2). The reaction is simply performed by grinding the reagents, thus allowing for minimal use of solvents, which are employed only in small quantities to extract the product in the final step. It is well reported that this procedure affords, in situ, the Au-OH species, which can subsequently react with organic acidic substrates, such as phenylacetylene [12,19]. In our case, this procedure was successful only for the complexes with a linker longer than one methylene between the carbene units. This could be due to the use of the strong base during synthesis; for example, it is well documented in the literature that the treatment of bis(imidazolium) salts bearing a CH_2_ linker with a strong base and a metal precursor does not allow for a straightforward synthesis of the corresponding metal complexes [20,21]. In the literature, dinuclear alkynyl complexes with a CH_2_ linker between the carbene moieties were isolated either through the reaction of the [(diNHC)(AuBr)_2_] complex with K_2_CO_3_ and phenylacetylene in methanol at room temperature or through the transmetalation of the diNHC ligand to the polymeric gold(I) phenylacetylide [12,17].

**Figure 1:**
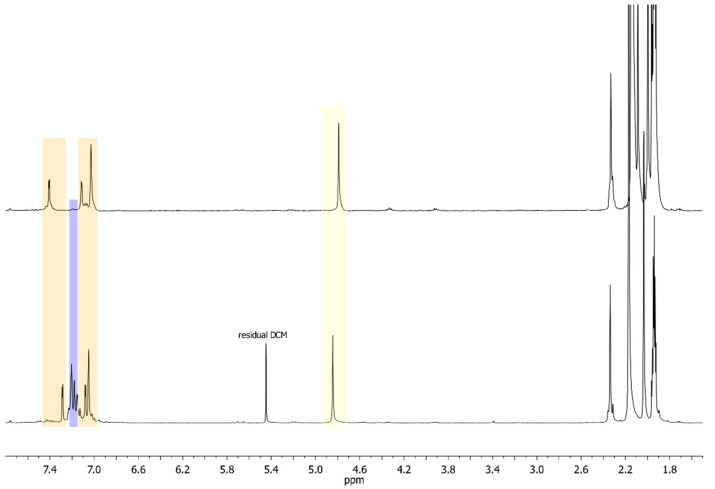
Comparison of the ^1^H-NMR spectrum of complex **5** (lower spectrum) with the pristine dibromido complex **2** (upper spectrum).

The formation of complexes **5**–**7** was confirmed through ^1^H-NMR analysis: a multiplet is present at ca. 7.2 ppm, attributed to the phenyl protons, integrating for about 10H (see, for example, Figure 1, where the signals are highlighted in light purple). The signals associated with the diNHC ligand are characterized only by a small shift going from -Br to -C≡CR derivatives (see Figure 1 and compare the two spectra, in the yellow regions, which represent the hydrogens of the ethylene bridge, or in the orange regions, which represent the aromatic protons of the diNHC ligand). Furthermore, the similarity of the spectra suggests that the symmetric structure of the complex is maintained during the reaction, i.e., both gold centers are characterized by the same coordination environment.

The formation of the alkynyl complex is also supported by the ^13^C-NMR spectra, in which the carbene carbon signal is located at 180–185 ppm, shifted upfield from the corresponding signal in the starting [(diNHC)(AuBr)_2_] complex. Finally, the main evidence of the successful reaction comes from the mass spectra, in which the main signal is associable with the [(diNHC)Au_2_(C≡CPh)]^+^ fragment (*m*/*z* 893, 907, and 991 for complexes **5**, **6**, and **7**, respectively). Unfortunately, despite several attempts, we were not able to obtain crystals of complexes **5**–**7** suitable for X-ray diffraction analysis.

**Scheme 3:**
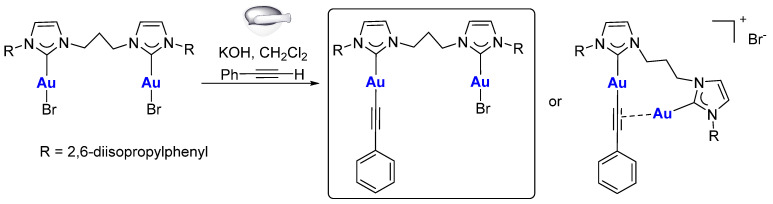
Synthesis of complex **8**.

**Figure 2:**
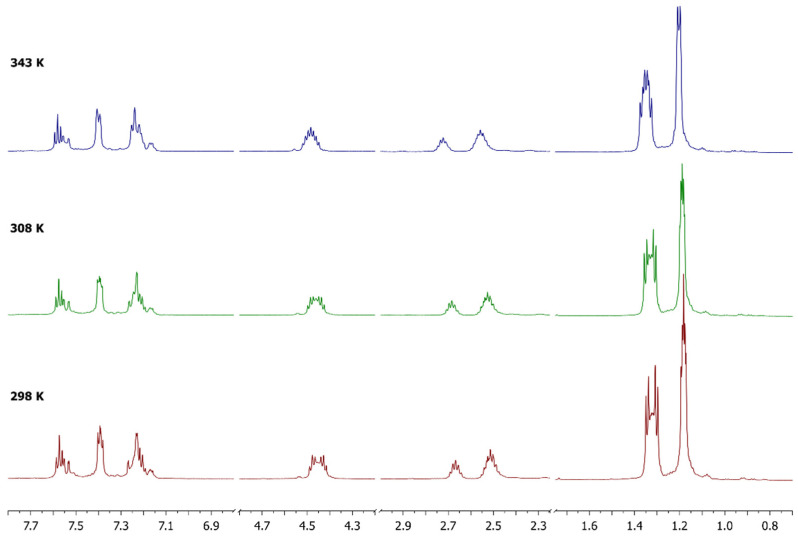
^1^H-NMR spectra of complex **8** in CD_3_CN at different temperatures.

Interestingly, in our preliminary evaluation of this synthetic procedure, we isolated a different product, complex **8**, characterized by the presence of only one phenylacetylide ligand (Scheme 3). The experimental procedure was similar to that used for the synthesis of complexes **5**–**7**, but using a lower amount of phenylacetylene (Au:PhC≡CH ratio 1:2). In the ^1^H-NMR spectrum of complex **8** (Figure 2), the signals of the diNHC ligand suggest a lower symmetry for the isolated species compared to the spectrum of complex **3**, as evident from the comparison between the multiplet at ca. 4.4 ppm that is associable with the NCH_2_ hydrogens of the bridge. Furthermore, the integration of the signals of the aromatic region is in agreement with the presence of only one phenylacetylide group.

VT-NMR experiments were performed within the range of 298–343 K (Figure 2) without observing coalescence of the signals, but with only small changes in the chemical shift and line broadening.

**Figure 3:**
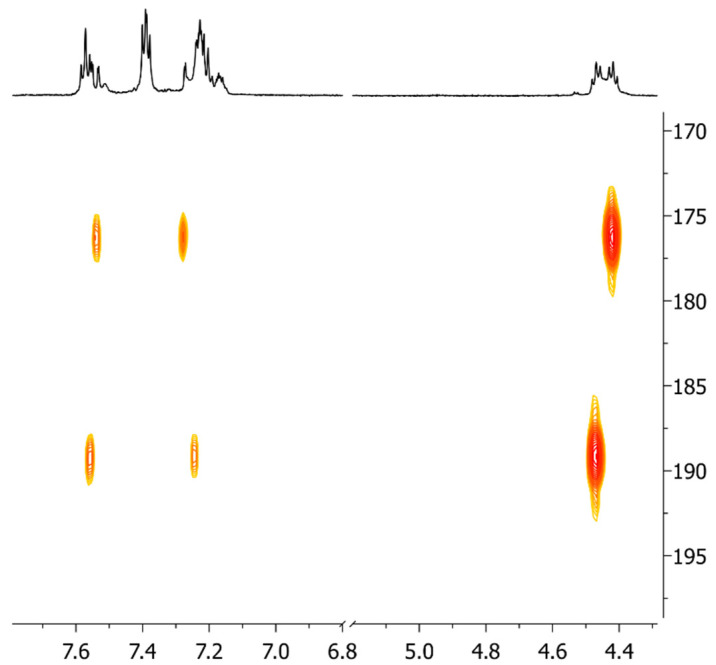
Detail of the ^1^H,^13^C-HMBC-NMR spectrum of complex **8** in CD_3_CN: cross-peaks involving the carbene carbons.

**Figure 4:**
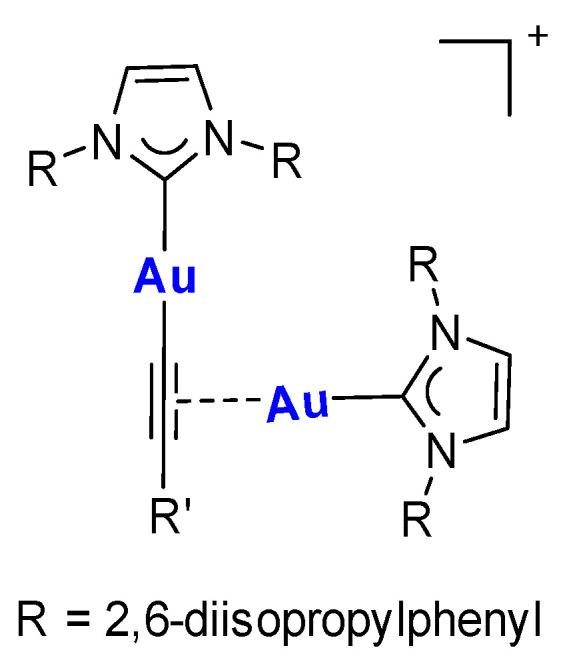
Structure of [IPr_2_Au_2_(C≡CR’)]^+^ type complexes reported in [22,23].

The ^1^H,^13^C-HMBC-NMR spectrum (Figure 3) also shows the presence of two different signals for the carbene carbons at 176.5 and 189.4 ppm. This suggests that the two gold(I) centers have different coordination spheres. The structures that can justify the experimental data are reported in Scheme 3: one with an acetylide ligand coordinated to one gold center and a bromido ligand to the other or one in which the acetylide moiety coordinates both gold(I) centers in a (σ, π) fashion. The ESI mass spectrum is not diagnostic, since it presents a signal at 991 *m*/*z* associable with a [(diNHC)Au_2_(C≡CPh)]^+^ fragment, which is also detected for the disubstituted complex **7**. Nevertheless, the NMR data and, in particular, the chemical shifts of the carbene carbons, are in agreement with the first proposed structure. In fact, in the literature, a (σ, π) coordination of alkyne was reported between two [Au(IPr)]^+^ fragments (Figure 4), but the chemical shifts of the two carbene carbons were not distinguishable [6,22,23]. This is not surprising, and the same behavior has also been observed for complexes bearing phosphines instead of NHC ligands: the two phosphorous atoms coordinated to the two gold centers having the (σ, π) alkyne present a unique signal in the ^31^P-NMR spectrum [24].

**Figure 5:**
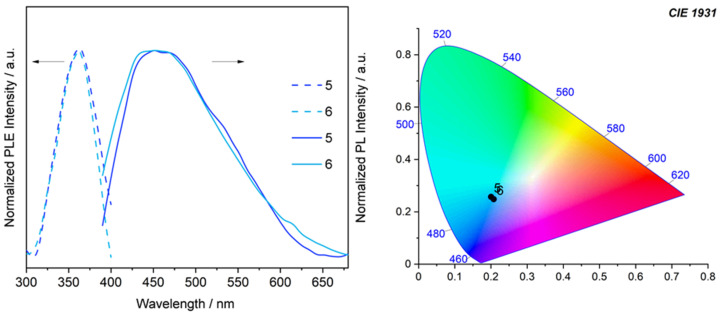
On the left, room temperature emission (solid line, λ_exc_ 360 nm) and excitation (PLE, dashed line) spectra of complexes **5** and **6** in the solid state. On the right, a CIE 1931 chromaticity diagram for complexes **5** and **6**.

Finally, the luminescence properties of [(diNHC)(AuC≡CPh)_2_] complexes **5** and **6**, differing in the length of the bridge between the carbene moieties, were investigated in the solid state at room temperature and compared with those of some analogous dicarbene alkynyl complexes reported in the literature [12,17]. The two analyzed complexes show a broad emission covering almost all the visible range centered at ca. 460 nm (Figure 5); the complexes’ emissions are shown in the blue–white region of the CIE 1931 chromaticity diagram (Figure 5). The photoluminescence excitation spectra of the two complexes are almost superimposable and lay in the UV region with a maximum of 360 nm. These data are in agreement with those reported in the literature for analogous phenylacetylide complexes. For example, the complexes [{RIm(CH_2_)_3_ImR}(AuC≡CPh)_2_] (Im=imidazol-2-ylidene, R = Bu or Bn), in the solid state at 298 K, show comparable band shape and Stokes shift, i.e., the emission profile is broad and centered at ca. 480 nm when excited at 350 nm [17]. Analogously, dinuclear complexes [{BnBIm(CH_2_)_3_BImBn}(AuC≡CPh)_2_] with benzimidazole-based (BIm) dicarbene ligands show the same behavior [12]. In the literature, the emission in the complexes [(diNHC)(AuC≡CPh)_2_] is identified as phosphorescence, which is associated with an intraligand transition involving the C≡CPh fragments perturbed by the metal center [12,17]. Phosphorescence also seems operative in our complexes, considering that the emission lifetime of complex **5** is close to 14 μs. Furthermore, the position of the emission band and the comparison with the data reported in the literature suggest the absence of a contribution of the intramolecular aurophilic interactions in the determination of the emitting properties [12]. This is not surprising if we consider the X-ray crystal structures of the pristine bromido complexes **2** and **3** and their Au···Au distances (5.14 and 4.08 Å, respectively).

## 3. Materials and Methods

### 3.1. General Comments

All the commercially available reagents were used as received without additional purification. Complexes **1**–**4** were prepared according to procedures established in the literature [25]. The NMR spectra were usually recorded on a Bruker Avance 300 (300.1 MHz for ^1^H and 75.5 MHz for ^13^C) at 298 K; the spectra of complex **8** were also recorded on a Bruker Avance DMX-600 MHz spectrometer operating at 599.90 MHz for ^1^H and 150.61 MHz for ^13^C, respectively. The temperature was controlled (±0.1 °C) with a Bruker BVT-3000 temperature controller; chemical shifts (δ) are reported in units of ppm relative to the residual solvent signals. ESI-MS analyses of the new compounds were performed using an LCQ-Duo (Thermo-Finnigan, San Jose, CA, USA) operating in positive ion mode, dissolving the compounds in acetonitrile, and directly infusing the prepared solution into the ESI source using a syringe pump.

### 3.2. General Procedure for the Reaction between the [(diNHC)(AuBr)_2_] Complexes with Phenylacetylene

In a nitrogen-filled glove box, a pellet of KOH (ca. 100 mg, 2.0 mmol) was powdered in a mortar until a fine white powder was obtained. Phenylacetylene (100 μL, 1.0 mmol) was then added, followed by the proper [(diNHC)(AuBr)_2_] complex (0.05 mmol) and 2 mL of dichloromethane. Grinding was continued for 5–10 min, observing the formation of an orange solid. The mixture was treated with dichloromethane (3 × 5 mL), separating the organic phase from the solid each time. Finally, the organic solution was filtered on a syringe with a PTFE 0.45 μm filter, and the solvent was evaporated to dryness. The off-white residue was washed with 15 mL hexane, filtered, and dried under vacuum.

Complex **5**. ^1^H-NMR (DMSO-*d*_6_, 300 MHz, ppm) δ: 1.99 (s, 6H, CH_3_), 2.30 (s, 12H, CH_3_), 4.85 (s, 4H, CH_2_), 7.05 (s, 4H, CH Mes), 7.17–7.18 (m, 10H, CH Ph), 7.46 (d, ^3^J_HH_ = 1.2 Hz, 2H, 4-Im), 7.61 (d, ^3^J_HH_ = 1.2 Hz, 2H, 5-Im). ^13^C{^1^H}-NMR (DMSO, 75 MHz, ppm) δ: 18.0 (CH_3_), 20.7 (CH_3_), 50.8 (CH_2_), 103.7 (*C*≡CAu), 121.9–138.7 (aromatic CH and C carbons, 4,5-Im, and C≡*C*Au), 186.3 (2-Im). ESI-MS (positive mode, CH_3_CN): *m/z* 893 [(diNHC)Au_2_(C≡CPh)]^+^.

Complex **6**. ^1^H-NMR (CD_3_CN, 300 MHz, ppm) δ: 2.06 (s, 12H, CH_3_), 2.34 (s, 6H, CH_3_), 2.59 (m, 2H, CH_2_), 4.40 (m, 4H, CH_2_), 7.06 (s, 4H, CH Mes), 7.11 (d, ^3^J_HH_ = 1.2 Hz, 2H, 4-Im), 7.15-7.18 (m, 10H, CH Ph), 7.48 (d, ^3^J_HH_ = 1.2 Hz, 2H, 5-Im). ^13^C{^1^H}-NMR (CD_3_CN, 75 MHz, ppm) δ: 18.1 (CH_3_), 21.1 (CH_3_), 32.2 (CH_2_), 48.9 (CH_2_), 104.3 (*C*≡CAu), 122.3–140.4 (aromatic CH and C carbons, 4,5-Im, and C≡*C*Au), 188.1 (2-Im). ESI-MS (positive mode, CH_3_CN): *m/z* 907 [(diNHC)Au_2_(C≡CPh)]^+^.

Complex **7**. ^1^H-NMR (CD_3_CN, 300 MHz, ppm) δ: 1.14 (d, ^3^J_HH_ = 6.0 Hz, 12H, CH_3_), 1.29 (d, ^3^J_HH_ = 6.0 Hz, 12H, CH_3_), 2.45 (m, 4H, CH iPr), 2.62 (m, 2H, CH_2_), 4.43 (m, 4H, CH_2_), 7.14–7.20 (m, 4-Im and CH Ph), 7.34–7.37 (m, 6H, CH Ar), 7.51 (d, ^3^J_HH_ = 1.8 Hz, 2H, 5-Im). ^1^H NMR (DMSO, 300 MHz, ppm) δ: 1.12 (d, ^3^J_HH_ = 6.0 Hz, 12H, CH_3_), 1.25 (d, ^3^J_HH_ = 6.0 Hz, 12H, CH_3_), 2.38 (m, 4H, CH iPr + CH_2_), 4.42 (m, 4H, CH_2_), 7.11–7.21 (m, 10H, CH Ph), 7.36–7.52 (m, 6H, CH Ar), 7.59 (d, ^3^J_HH_ = 1.8 Hz, 2H, 4-Im), 7.89 (d, ^3^J_HH_ = 1.8 Hz, 2H, 5-Im). ^13^C{^1^H}-NMR (DMSO, 75 MHz, ppm) δ: 23.6 (CH_3_), 24.0 (CH_3_), 29.5 (CH iPr), 32.6 (CH_2_), 52.0 (CH_2_), 106.7 (*C*≡CAu), 123.6–145.7 (aromatic CH and C carbons, 4,5-Im, and C≡*C*Au), 182.6 (2-Im). ESI-MS (positive mode, CH_3_CN): *m/z* 991 [(diNHC)Au_2_(C≡CPh)]^+^.

Complex **8**. Same procedure reported for complexes **5**–**7**, but using 20 μL (0.2 mmol) of phenylacetylene. ^1^H-NMR (CD_3_CN, 600 MHz, ppm) δ: 1.14 (m, 12H, CH_3_), 1.28 (m, 12H, CH_3_), 2.46 (m, 4H, CH iPr), 2.61 (m, 2H, CH_2_), 4.41 (m, 4H, CH_2_), 7.14–7.55 (m, 15H, 4-Im, 5-Im, CH Ar, and CH Ph). ^13^C-NMR (CD_3_CN, 150 MHz, ppm) δ: 24.4 (CH_3_), 24.6 (CH_3_), 29.3 (CH iPr), 33.3 (CH_2_), 49.2 (CH_2_), 104.7 (*C*≡CAu), 122.4–147.2 (aromatic CH and C carbons and 4,5-Im), 176.3 (2-Im), 189.2 (2-Im). ESI-MS (positive mode, CH_3_CN): *m/z* 992 [(diNHC)Au_2_(C≡CPh)]^+^.

### 3.3. Luminescence Measurements

The luminescence spectra on powders were recorded at room temperature using a Fluorolog-3, Horiba JobinYvon spectrofluorimeter equipped with a double-grating monochromator on both the excitation and emission sides. A 450 W Xe arc lamp and an R928P Hamamatsu photomultiplier were employed as an excitation source and detector, respectively. The emission spectra were corrected for detection and optical spectral response of the spectrofluorimeter supplied by the manufacturer. Emission lifetimes were recoded with the same instrument using a 295 nm pulsed LED (pulse duration of a few ns) as an excitation source.

## 4. Conclusions

Above, we presented a simple method for isolating dinuclear gold(I) complexes with one di(N-heterocyclic carbene) ligand bridging the two metal centers and one phenylacetylide ligand per metal center to complete their coordination sphere. Mechanochemistry allows the reaction to be performed in a short amount of time with minimal work up. These gold compounds were fully characterized, and their solid-state luminescence properties were also investigated: the complexes emit in the blue–white region of the CIE 1931 chromaticity diagram. Work extending this approach to other alkynes and examining the possible applications of these complexes is ongoing.

## Data Availability

Not applicable.

## Sample Availability

Samples of the compounds are available from the authors.
